# Supplementary material for: Direct and indirect entomological efficacy of Targeted Indoor Residual Spraying against Aedes aegypti in Iquitos, Peru
Source: PLoS Negl Trop Dis. 2026 Oct 21;20(10):e0014390. doi: 10.1371/journal.pntd.0014390 (PMC13712745; doi:10.1371/journal.pntd.0014390)
Supplement: Supplementary file 2 [file pntd.0014390.s001.docx]

**Table S1.** Results from World Health Organization cone bioassays carried out on four wall surface types (concrete, brick, wood, and plywood) sprayed with a recommended dose of 1gr/m^2^ of Actellic 300CS with IK-Vector Super (Goizper Group, Spain) with 8002E nozzle. Cones were placed at 0.5, 1, and 1.5 m on each surface type 24 hours post insecticide application on treated and untreated surfaces. Ten mosquitoes were placed in each coned and knockdown was accessed at 30 minutes. All mosquitoes were then transferred from the cones to pint cardboard cups, placed inside a Styrofoam cooler and provided and provided cotton balls soaked with a 10% sucrose solution. Mortality 24 hours post-exposure was accessed.

| Time after spray | Sprayed w/ pirimiphos-methyl 30% | | Control (unsprayed) | |
| --- | --- | --- | --- | --- |
|  | *Aedes aegypti*  females (n) | 24 h (%) | *Aedes aegypti*  females (n) | 24 h (%) |
| 24 hours  Concrete  Brick  Wood  Plywood | 60  60  60  60 | 87  85  100  100 | 60  60  60  60 | 0  0  0  0 |
| 1 months  Concrete  Brick  Wood  Plywood | 60  60  60  60 | 75  33  97  100 | 60  60  60  60 | 0  0  0  0 |
| 5 months  Concrete  Brick  Wood  Plywood | 30  30  30  30 | 0  3  0  7 | 30  30  30  30 | 7  0  0  10 |
