# Supplementary material for: Direct and indirect entomological efficacy of Targeted Indoor Residual Spraying against Aedes aegypti in Iquitos, Peru
Source: PLoS Negl Trop Dis. 2026 Oct 21;20(10):e0014390. doi: 10.1371/journal.pntd.0014390 (PMC13712745; doi:10.1371/journal.pntd.0014390)
Supplement: Supplementary file 3 [file pntd.0014390.s002.docx]

**Table S2.** Results from Generalized Linear Mixed models (GLMM) testing the difference between arms (control as baseline versus TIRS) in the number of *Ae. aegypti* per house for the direct efficacy dataset for each of the four entomological survey conducted post-spraying. Cluster ID was used as random intercept.

| **Time since TIRS** | **Parameter** | **Estimate** | **Std. Error** | **Z-value** | **P** |
| --- | --- | --- | --- | --- | --- |
| 1-15 days | Intercept | 0.556 | 0.228 | 2.44 | 0.0148 |
|  | Treatment Arm | -3.349 | 0.416 | -8.05 | <0.001 |
| 30-45 days | Intercept | 0.333 | 0.121 | 2.74 | 0.006 |
|  | Treatment Arm | -3.109 | 0.267 | -11.63 | <0.001 |
| 180-195 days | Intercept | -0.205 | 0.150 | -1.36 | 0.17 |
|  | Treatment Arm | -0.548 | 0.226 | -2.43 | 0.015 |
| 240-255 days | Intercept | 0.122 | 0.123 | 0.99 | 0.32 |
|  | Treatment Arm | -0.533 | 0.187 | -2.85 | 0.004 |
