# Supplementary material for: Direct and indirect entomological efficacy of Targeted Indoor Residual Spraying against Aedes aegypti in Iquitos, Peru
Source: PLoS Negl Trop Dis. 2026 Oct 21;20(10):e0014390. doi: 10.1371/journal.pntd.0014390 (PMC13712745; doi:10.1371/journal.pntd.0014390)
Supplement: Supplementary file 4 [file pntd.0014390.s003.docx]

**Table S3.** Results from Generalized Linear Mixed models (GLMM) testing the difference between arms (control as baseline versus TIRS) in the number of *Ae. aegypti* per house for the indirect efficacy dataset for each of the four entomological survey conducted post-spraying. Cluster ID was used as random intercept.

| **Time since TIRS** | **Parameter** | **Estimate** | **Std. Error** | **Z-value** | **P** |
| --- | --- | --- | --- | --- | --- |
| 1-15 days | Intercept | 0.570 | 0.111 | 5.11 | <0.001 |
|  | Treatment Arm | -1.179 | 0.249 | -4.73 | <0.001 |
| 30-45 days | Intercept | 1.520 | 0.050 | 30.10 | <0.001 |
|  | Treatment Arm | -1.083 | 0.078 | -14.01 | <0.001 |
| 180-195 days | Intercept | -0.207 | 0.154 | -1.34 | 0.17 |
|  | Treatment Arm | -0.234 | 0.250 | -0.94 | 0.35 |
| 240-255 days | Intercept | 0.111 | 0.147 | 0.75 | 0.45 |
|  | Treatment Arm | -0.076 | 0.241 | -0.31 | 0.75 |
