# Supplementary material for: Direct and indirect entomological efficacy of Targeted Indoor Residual Spraying against Aedes aegypti in Iquitos, Peru
Source: PLoS Negl Trop Dis. 2026 Oct 21;20(10):e0014390. doi: 10.1371/journal.pntd.0014390 (PMC13712745; doi:10.1371/journal.pntd.0014390)
Supplement: Supplementary file 5 [file pntd.0014390.s005.docx]

Reviewer Responses (Editor comments)

My overall assessment of provided reviews is that reviewers see value in the work and find the work technically sound. Only minor comments are provided - mostly along the lines of added inference (such as information on IR, costs of TIRS, flexibility of TIRS to other products); the other other minor comments related to word choice, acronym definitions, and sentence structure. In addition to these reviews, I have minor suggestions/comments related to provided Tables and Figures:

*Response:* *See are responses to the reviewer comments where we address the reviewer comments and added text to the manuscript.*

Table 3 - it would be stronger to back-transform the model coefficients so that the table actually reflects what baseline estimates are. It also appears these estimates may already be listed in Table 1 (perhaps these are the raw calculations?). Table 3 is maybe not needed as a main table.

*Response: The values listed in table 1 are the actual percentages. We now used the model to estimate the number of Ae. aegypti per house (and its 95%CI) after accounting for heterogeneity within houses. The table now includes the following: “Back-transformed Number of Ae. aegypti per house by arm (95%CI) was: 1.94 (1.49-2.53) for control and 2.44 (1.86-3.19) for TIRS.”*

Figure 3 - I'm assuming the whiskers are standard errors? If so, there is an error in Panel A as one whisker shows negative mosquitoes as a possibility.

*Response. The values are 95%CI. We re-calculated them using binomial counts to avoid the negative values.*

Tables 4 & 5 - the manuscript is short & likely doesn't need supporting info. However, Tables 4/5 (& Table 3) just appear to be raw GLMM tables - average readers may not find these very appealing. Some of this info is already in Figure 3 and 4. It might be worth while to push raw GLMM tables to supporting, since figures/main research tables capture the important information being conveyed.

*Response:* *We moved the tables to supplement. Table 4 is now Table S2. Table 5 is now Table S3.*

Figure 4 - since GLMM outputs are in discrete temporal windows of back checks, how are linear connections among points extrapolated? Provided methods are insufficient to determine this.

*Response:* *This is a simple LOESS smoothing of the data. We now provide this on the base of the table. Given this is a fairly standard method, we did not include it in the methods, as it was only used for improving visualization.*

Please address these comments carefully as you prepare a revised manuscript, and be sure to provide a track-changes document in addition to your clean version and point-by-point responses once a resubmission is prepared.

Reviewer #1: Comments on: Direct and Indirect Entomological Efficacy of Targeted Indoor Residual Spraying against Aedes aegypti in Iquitos, Peru.
This manuscript adds support to previous ones conducted in other countries on the use of TIRS. The reported work is clearly written, with appropriate sample sizes, experimental design, statistical analyses, and conclusions.

*Response: Thank you for your kind words.*

Some information about previous knowledge on insecticide resistance against pirimiphos-methyl would be useful to better understand the results.

*Response:* Very *appropriate question, and unfortunately, we were not able to conduct either WHO or Bottle Assays at the time of the experiment because we did not have access to technical grade pirimiphosmethyl to conduct the assays. We did however, conduct WHO cone biosassays* prior to our experiment that show high mortality 24 hours after application, but that residual efficacy decreased overtime depending on the wall surface. **SEE Lines 232-237 and add S1 Table.** P*rior to our trial, pirimiphosmethyl was only used for Anopheles control in rural communities outside Iquitos. For this reason expected Aedes aegypti would be susceptible. After consultation with our MOH colleagues, they claim that Ae. aegypti are susceptible to both malathion and pirimiphosmethyl based on WHO tests showing mortality above 80%.*

How general is this insecticide used in the region?

*Response:*  *Used more in Africa, but it is the new state of the art product (not that new anymore) for malaria control, but never used for Aedes aegypti in Peru. Worldwide this is one of the most used chemicals in malaria endemic regions.*

It does not seem to be authorized in Europe or the US.

*Response: See added text lines 524-527. Our understanding is that it is not worth it to chemical companies to get EPA approval for an Indoor Residual spray, because IRS is not needed or popular in the US. We don’t have many places in the US with endemic malaria transmission. This is an important issue for the military as they will not use products without EPA approval, which does not make sense in endemic regions.*

Any reason for not been authorized in those countries? (*See previous comment)*

Did you observe any adverse effects? *We did not, in fact people gave very positive feedback, clearly killed insects and no side affects.*

For how long would you recommend continuing spraying with pirimiphos-methyl? One year, two years, etc.?

*Response: See added text in Lines 484-490 where we emphasize that any insecticide campaign done for a single year will not be effective. Modeling studies (Hladish et al. PLoS NTD 2018, Hladish et al. PNAS 2020) show TIRS effectiveness would accumulate over time, particularly at high coverage. So, a single application in a single year is likely to have a limited impact in reducing dengue burden. TIRS is another tool in the toolbox for vector control programs.*

Any alternative insecticide to prevent resistance?
What other insecticides could be used instead of pirimiphos-methyl, so that it can be acceptable everywhere?

*Response: There are several studies that have compared efficacy in field conditions or experimental houses of at least two additional insecticides. Chlorfenapyr (Che-Mendoza et al. PLoS Negl Trop Dis. 2021 Oct 4;15(10):e0009822) and also clothianidine (Che-Mendoza et al. Pest Manag Sci. 2023 Feb;79(2):638-644).*

Do authors have data on the public health impact of their work, such as on dengue during the study?

*Response: No this study was only funded for entomological indicators, but our group have carried out multiple vector control interventions in Iquitos, which require multiple years, huge costs and a little luck to have a properly powered dengue trial. For TIRS, co-author Vazquez-Prokopec has been conducting a clinical trial in Merida Mexico which we reference in the text. He is currently publishing data from secondary outcomes (seroconversions) which are more encouraging than those of the primary outcome (dengue cases). This trial was carried out when DENV transmission was relatively low inside Iquitos, so even if we had monitored the human population there would not have been sufficient transmission to measure a public health impact. That being said, years of evaluation of space sprays in Iquitos showed that when coverage is high (coverage rates are not as high as when pyrethroids were available), and you can sustain large reductions (>90%) for at least 3 weeks, transmission could be significantly mitigated. Again we discuss this in the current manuscript.*

Compared with other methods to control Aedes aegypti, do you have any idea of the comparative cost of TIRS?

*Response: We have added some text in Lines 397-406 to directly address the issue of costs. It is an important issue and the reviewer is correct to bring it up but getting accurate estimates has been challenging. We have been provided with reports from our MOH colleagues on the costs of space spray campaigns which illustrate that Public Health Programs spend a lot of money on these campaigns when they are not viewed as that effective. Others have published on this issue that often these campaigns are driven more by politics than science. Insecticide costs are very complicated and the subject of many corruption scandals. Procurement of insecticides is a complaint from many of our colleagues in vector control, but not something anyone wants to talk about publicly, but it complicates getting hard numbers on insecticide costs. For this trial, the Actellic was donated to us and was probably donated to the MOH or was subsidized is some form, but we were unable to get reliable information here.* Vazquez-Prokopec et al. (*Sci Rep* **12**, 21998 (2022)), *compared costs in Mexico and is now referenced and discussed in more detail, but it also makes a lot of assumptions. In Iquitos, we have seen from 1-3 space spray campaigns per year and it is difficult to imagine a preventative TIRS campaign, what struck our research team was how much more effective TIRS was at accomplishing the aims of a 3 cycle space spray campaign, and just considering a single cycle strategy (albeit taking more time) compared to 3 cycles seemed like costs should be competitive without considering the insecticide costs. We sincerely, hope this publication will convince decision makers in Peru to consider TIRS, but they need to cost it out.*

Reviewer #2: The manuscript titled “Direct and Indirect Entomological Efficacy of Targeted Indoor Residual Spraying against Aedes aegypti in Iquitos, Peru” evaluates the effectiveness of the TIRS method in reducing indoor mosquito densities and, consequently, the potential risk of dengue transmission in an urban setting. The authors assessed the direct versus indirect impact of TIRS by aspirating resting mosquitoes before the intervention and at three post intervention time points over an eight-month period.
Direct and indirect efficacy in reducing mosquito densities was 96% vs 69% at 15 days post-intervention, and 40% vs 7% at 255 days. They conclude that both direct and indirect TIRS effect produces substantial reductions in mosquito density during early post intervention surveys, whereas late post intervention effects are observed only in directly treated clusters.

This study is relevant for vector control programs, and the authors appropriately recommend TIRS for both preventive and reactive applications aimed at achieving substantial and sustained reductions in indoor Ae. aegypti densities. Overall, the manuscript is well written, and the statistical analyses adequately support the reported efficacy of the intervention across both directly and indirectly treated clusters. I recommend accepting it with minor revisions.

Minor revision
Introduction
L-78. Describe what IRS means. *Spelled out Indoor residual spray when first used)*
L-87. Delete “indoor residual spaying” and just leave “IRS”. *done*
Methods
L-192. Describe what MINSA means. *Ministry of Health (MOH), donde*
Discussion
About the last statement, description of three acronyms has not been pronounced before in the manuscript, that might make it a bit challenging for readers who aren’t familiar with these terms.

*Response: now spelled out: Existing Integrated Pest-- and Endemic Vector – management plans*

Since insecticide resistance is important for vector control and it is mentioned twice in the Discussion, it would be helpful to include one or two references showing how resistance can diminish post spray reductions in mosquito density. This would reinforce the point and improve the scientific grounding of the section.

*Response: We added the following sentence on Results line 211. “In 2014, Iquitos Ae. aegypti was shown to be resistance to pyrethroids greatly complicating vector control in the city [43,50].”*

Reviewer #3: Strong study overall, great to see advancements in Aedes control beyond indoor space spray are being further validated in field trials and provide longer population suppression.

Reviewer Responses

From Attached document (Reviewer #3)

1. Epidemics of dengue? Why does this affect control? I recommend removing this from abstract unless can be expanded/explained

*Response:*  *Sentence has been modified as follows (Ln 29-30): …”* existing tools, and that control activities often occur in the context of large dengue epidemics that overwhelm control programs”.

2. Clarify that this is reduction prokopack aspiration counts

*Response:*  *Sentence has been modified as follows (line 44) with added text in bold:* Direct efficacy reached a 96% decrease **in *Ae. aegypti* captured in sprayed houses** at 15 days post-spraying.

3. this sentence diminishing the importance of vector control measures for control of dengue. The vaccine is not available yet so we still need the best vector control methods.

*Response: We are a bit confused by this comment and its placement in the manuscript, since this text does not currently exist in the manuscript. Our interpretation is that you want the second sentence added which is an important point. Since this project was funded by the DOD, the authors are obligated to discuss the impacts on military populations. Please see added text in Lines 73-75, shown below in bold.*

*…* multiple DENV serotypes [5]. **Thus, vector control remains critical to prevent dengue because current vaccines protect primarily against secondary infections, dengue vaccines are not an option for** service members who often experience more frequent exposure to disease vectors due to time spent in austere environments participating in activities such as field exercises,

4. *spell out this acronym (HDRV)*

*Response: We now spell out this acronym (Ln 77-78): “*Humanitarian Assistance and Disaster Relief (HADR) operations”

5. Are aerial applications the only tool available? Why specifically bringing this up? Nothing in the intro supports that aerial application are the best mode to control indoor resting Ae aegypti. Recommend omitting this or building better argument why this is a concern that justifies the need for targeted IRS.

*Response: Happy to remove in the introduction, this comment is better suited for the discussion. The point being made is DOD often uses aerial spraying despite the lack of evidence (note the response is coming from one of the civilians on the author line). Any way thanks for the observation providing us justification to remove this sentence.*

6. close the parenthesis *-🡪 done (line 165)*
7. can larvicide be listed? Bti? Pyriproxyfen? *-🡪 done added pyriproxyfen (Line 167)*
8. Was malathion sprayed in every structure in each cluster.

*Response: No, but this was a Ministry of Health (MOH) Emergency Control Activity so we don’t have their data to say what their coverage rates are which are generally low (55% maximum in our experience).* *Ironically, our group has carried out multiple vector control trials in Iquitos and in almost every case we have to document their interventions the best we can. We had to scramble and coordinate with the MOH team to conduct before and after surveys in areas that were sprayed and not yet sprayed so we could try to estimate the impact, which are results clearly show were negligible. My guess is coverage was not good, but it is probably consistent across clusters.*

9. Is this a trade marked software? please define*. -🡪 added reference. QGIS (Quantum Geographic Information System), but known as QGIS and citation advice asks to refer to it as QGIS.*
10. Please write out/define this acronym -🡪 *Changed to Ministry of Health (MOH) (Line 194)*
11. What is HA? -🡪 *Helvio Astete the lead author. I have used this approach in previous PLOS articles, although not necessary it does indicate the role.*
12. Is this a subsample, How many? A percentage of the total? 🡪 Line 228

*Response: Truth is this was a convenience subsample. There was a maximum that the team was able to process each day and in general the team did not have time to score mosquitoes for afternoon collections. However, after discussion with the lead author we have decided to remove this sentence from the manuscript because we do not present this data further because it is always difficult to dissect and score sufficient mosquitoes to say anything meaningful and we did not observe anything insightful. I think our rational for keeping it was to say we did the work, but it does not add anything so we will remove.*

13. Was it only 1.5 m up the door frame or the entire door and window frame?

*Response: All surfaces include doors and window frames. See modified text (line 243-244)*

“The insecticide was applied on exposed surfaces at a height below 1.5 m including doors and window frames, and below and behind furniture.”

14. Could this be a percentage change in general over time and include the control values? Would be nice to show if populations tended to increase when no application was conducted, or dip slightly.

*Response: Excellent point and we have done this for presentations, not sure what were thinking data has been added to Table 1.*

15. list the number of days post application for each POST period 🡪 *see modified Table*
16. pure insecticide or diluted in water? 🡪 *diluted and added to text.*
17. Hand held? *Yes and added to text.*
18. I think this is a typo? Shoud be “at a larger scale” -🡪 *Yes and corrected.*
19. Remove “s” -🡪 done
20. Spelling -🡪 done.
