# Supplementary material for: Direct and indirect entomological efficacy of Targeted Indoor Residual Spraying against Aedes aegypti in Iquitos, Peru
Source: PLoS Negl Trop Dis. 2026 Oct 21;20(10):e0014390. doi: 10.1371/journal.pntd.0014390 (PMC13712745; doi:10.1371/journal.pntd.0014390)
Supplement: Peer review history

# Peer review: Decision Letter 0

**DOI:** 10.1371/journal.pntd.0014390.r001

**Authors:** Audrey Lenhart, Joseph McMillan

2 Sep 2026

Response to Reviewers Revised Manuscript with Track Changes Manuscript

Shaden Kamhawi

co-Editor-in-Chief

PLOS Neglected Tropical Diseases

orcid.org/0000-0003-4304-636XX

Paul Brindley

co-Editor-in-Chief

PLOS Neglected Tropical Diseases

orcid.org/0000-0003-1765-0002

**Additional Editor Comments (if provided):**

Table 3 - it would be stronger to back-transform the model coefficients so that the table actually reflects what baseline estimates are. It also appears these estimates may already be listed in Table 1 (perhaps these are the raw calculations?). Table 3 is maybe not needed as a main table.

Figure 3 - I'm assuming the whiskers are standard errors? If so, there is an error in Panel A as one whisker shows negative mosquitoes as a possibility.

Tables 4 & 5 - the manuscript is short & likely doesn't need supporting info. However, Tables 4/5 (& Table 3) just appear to be raw GLMM tables - average readers may not find these very appealing. Some of this info is already in Figure 3 and 4. It might be worth while to push raw GLMM tables to supporting, since figures/main research tables capture the important information being conveyed.

Figure 4 - since GLMM outputs are in discrete temporal windows of back checks, how are linear connections among points extrapolated? Provided methods are insufficient to determine this.

**Journal Requirements:**

**Reviewers' comments:**

**Key Review Criteria Required for Acceptance?**

As you describe the new analyses required for acceptance, please consider the following:

**Methods**

-Are the objectives of the study clearly articulated with a clear testable hypothesis stated?

-Is the study design appropriate to address the stated objectives?

-Is the population clearly described and appropriate for the hypothesis being tested?

-Is the sample size sufficient to ensure adequate power to address the hypothesis being tested?

-Were correct statistical analysis used to support conclusions?

-Are there concerns about ethical or regulatory requirements being met?

Reviewer #1: (No Response)

Reviewer #2: The objectives and hypothesis are clear stated

Design study was appropriated for the objectives as was the mosquito population collected to test the hypothesis

Sample size was sufficient

Statistics were correctly used

Authors met ethical requirements

Reviewer #3: No concerns, just some minor information needed, see attached with comments for the specifics.

**********

**Results**

-Does the analysis presented match the analysis plan?

-Are the results clearly and completely presented?

-Are the figures (Tables, Images) of sufficient quality for clarity?

Reviewer #1: (No Response)

Reviewer #2: The analysis presented match the plan

Results are clear and complete

Tables and images have quality

Reviewer #3: Just minor points of clarification listed in comments on the attached.

**********

**Conclusions**

-Are the conclusions supported by the data presented?

-Are the limitations of analysis clearly described?

-Do the authors discuss how these data can be helpful to advance our understanding of the topic under study?

-Is public health relevance addressed?

Reviewer #1: (No Response)

Reviewer #2: Conclusions are supported by the data

Limitations are described

Discussion includes how these data can be helpful to advance our understanding of the topic

Public health relevance is addressed

Reviewer #3: No major issues.

**********

**Editorial and Data Presentation Modifications?**

Use this section for editorial suggestions as well as relatively minor modifications of existing data that would enhance clarity. If the only modifications needed are minor and/or editorial, you may wish to recommend “Minor Revision” or “Accept”.

Reviewer #1: (No Response)

Reviewer #2: (No Response)

Reviewer #3: Accept after minor revision.

**********

**Summary and General Comments**

Use this section to provide overall comments, discuss strengths/weaknesses of the study, novelty, significance, general execution and scholarship. You may also include additional comments for the author, including concerns about dual publication, research ethics, or publication ethics. If requesting major revision, please articulate the new experiments that are needed.

Reviewer #1: Comments on: Direct and Indirect Entomological Efficacy of Targeted Indoor Residual Spraying against Aedes aegypti in Iquitos, Peru.

This manuscript adds support to previous ones conducted in other countries on the use of TIRS. The reported work is clearly written, with appropriate sample sizes, experimental design, statistical analyses, and conclusions.

Some information about previous knowledge on insecticide resistance against pirimiphos-methyl would be useful to better understand the results.

How general is this insecticide used in the region? It does not seem to be authorized in Europe or the US. Any reason for not been authorized in those countries?

Did you observe any adverse effects?

For how long would you recommend continuing spraying with pirimiphos-methyl? One year, two years, etc.? Any alternative insecticide to prevent resistance?

What other insecticides could be used instead of pirimiphos-methyl, so that it can be acceptable everywhere?

Do authors have data on the public health impact of their work, such as on dengue during the study?

Compared with other methods to control Aedes aegypti, do you have any idea of the comparative cost of TIRS?

Reviewer #2: The manuscript titled “Direct and Indirect Entomological Efficacy of Targeted Indoor Residual Spraying against Aedes aegypti in Iquitos, Peru” evaluates the effectiveness of the TIRS method in reducing indoor mosquito densities and, consequently, the potential risk of dengue transmission in an urban setting. The authors assessed the direct versus indirect impact of TIRS by aspirating resting mosquitoes before the intervention and at three post intervention time points over an eight-month period.

Direct and indirect efficacy in reducing mosquito densities was 96% vs 69% at 15 days post-intervention, and 40% vs 7% at 255 days. They conclude that both direct and indirect TIRS effect produces substantial reductions in mosquito density during early post intervention surveys, whereas late post intervention effects are observed only in directly treated clusters.

This study is relevant for vector control programs, and the authors appropriately recommend TIRS for both preventive and reactive applications aimed at achieving substantial and sustained reductions in indoor Ae. aegypti densities. Overall, the manuscript is well written, and the statistical analyses adequately support the reported efficacy of the intervention across both directly and indirectly treated clusters. I recommend accepting it with minor revisions.

Minor revision

Introduction

L-78. Describe what IRS means.

L-87. Delete “indoor residual spaying” and just leave “IRS”.

Methods

L-192. Describe what MINSA means.

Discussion

About the last statement, description of three acronyms has not been pronounced before in the manuscript, that might make it a bit challenging for readers who aren’t familiar with these terms.

Since insecticide resistance is important for vector control and it is mentioned twice in the Discussion, it would be helpful to include one or two references showing how resistance can diminish post spray reductions in mosquito density. This would reinforce the point and improve the scientific grounding of the section.

Reviewer #3: Strong study overall, great to see advancements in Aedes control beyond indoor space spray are being further validated in field trials and provide longer population suppression.

**********

PLOS authors have the option to publish the peer review history of their article (what does this mean?). If published, this will include your full peer review and any attached files.

If you choose “no”, your identity will remain anonymous but your review may still be made public.

**Do you want your identity to be public for this peer review?**  For information about this choice, including consent withdrawal, please see our Privacy Policy.

Reviewer #1: No

Reviewer #2: No

Reviewer #3: No

**Figure resubmission:**

After uploading your figures to PLOS’s NAAS tool - https://ngplosjournals.pagemajik.ai/artanalysis, NAAS will process the files provided and display the results in the "Uploaded Files" section of the page as the processing is complete. If the uploaded figures meet our requirements (or NAAS is able to fix the files to meet our requirements), the figure will be marked as "fixed" above. If NAAS is unable to fix the files, a red "failed" label will appear above. When NAAS has confirmed that the figure files meet our requirements, please download the file via the download option, and include these NAAS processed figure files when submitting your revised manuscript.

**Reproducibility:** -->To enhance the reproducibility of your results, we recommend that authors of applicable studies deposit laboratory protocols in protocols.io, where a protocol can be assigned its own identifier (DOI) such that it can be cited independently in the future. Additionally, PLOS ONE offers an option to publish peer-reviewed clinical study protocols. Read more information on sharing protocols at https://plos.org/protocols?utm_medium=editorial-email&utm_source=authorletters&utm_campaign=protocols-->?>

Attachment Submitted filename: PNTD-D-26-00966_reviewer.pdf

# Author comment: Author response to Decision Letter 1

**DOI:** 10.1371/journal.pntd.0014390.r002

27 Sep 2026

Attachment Submitted filename: Reviewer Responses14_9_2026.docx

# Editor report: Decision Letter 1

**DOI:** 10.1371/journal.pntd.0014390.r003

**Authors:** Audrey Lenhart, Joseph McMillan

30 Sep 2026

Dear Dr. Morrison,

We are pleased to inform you that your manuscript 'Direct and Indirect Entomological Efficacy of Targeted Indoor Residual Spraying against Aedes aegypti in Iquitos, Peru' has been provisionally accepted for publication in PLOS Neglected Tropical Diseases.

Before your manuscript can be formally accepted you will need to complete some formatting changes, which you will receive in a follow up email. A member of our team will be in touch with a set of requests.

Please note that your manuscript will not be scheduled for publication until you have made the required changes, so a swift response is appreciated.

IMPORTANT: The editorial review process is now complete. PLOS will only permit corrections to spelling, formatting or significant scientific errors from this point onwards. Requests for major changes, or any which affect the scientific understanding of your work, will cause delays to the publication date of your manuscript.

Should you, your institution's press office or the journal office choose to press release your paper, you will automatically be opted out of early publication. We ask that you notify us now if you or your institution is planning to press release the article. All press must be co-ordinated with PLOS.

Thank you again for supporting Open Access publishing; we are looking forward to publishing your work in PLOS Neglected Tropical Diseases.

Best regards,

Joseph McMillan, PhD

Academic Editor

PLOS Neglected Tropical Diseases

Audrey Lenhart

Section Editor

PLOS Neglected Tropical Diseases

Shaden Kamhawi

co-Editor-in-Chief

PLOS Neglected Tropical Diseases

orcid.org/0000-0003-4304-636XX

Paul Brindley

co-Editor-in-Chief

PLOS Neglected Tropical Diseases

orcid.org/0000-0003-1765-0002

***********************************************************

# Editor report: Acceptance letter

**DOI:** 10.1371/journal.pntd.0014390.r004

**Authors:** Audrey Lenhart, Joseph McMillan

Dear Dr. Morrison,

We are delighted to inform you that your manuscript, "Direct and Indirect Entomological Efficacy of Targeted Indoor Residual Spraying against Aedes aegypti in Iquitos, Peru," has been formally accepted for publication in PLOS Neglected Tropical Diseases.

We have now passed your article onto the PLOS Production Department who will complete the rest of the publication process. All authors will receive a confirmation email upon publication.

The corresponding author will soon be receiving a typeset proof for review, to ensure errors have not been introduced during production. Please review the PDF proof of your manuscript carefully, as this is the last chance to correct any scientific or type-setting errors. Please note that major changes, or those which affect the scientific understanding of the work, will likely cause delays to the publication date of your manuscript. Note: Proofs for Front Matter articles (Editorial, Viewpoint, Symposium, Review, etc...) are generated on a different schedule and may not be made available as quickly.

Soon after your final files are uploaded, the early version of your manuscript will be published online unless you opted out of this process. The date of the early version will be your article's publication date. The final article will be published to the same URL, and all versions of the paper will be accessible to readers.

For Research Articles, you will receive an invoice from PLOS for your publication fee after your manuscript has reached the completed accept phase. If you receive an email requesting payment before acceptance or for any other service, this may be a phishing scheme. Learn how to identify phishing emails and protect your accounts at https://explore.plos.org/phishing.

Thank you again for supporting open-access publishing; we are looking forward to publishing your work in PLOS Neglected Tropical Diseases.

Best regards,

Shaden Kamhawi

co-Editor-in-Chief

PLOS Neglected Tropical Diseases

Paul Brindley

co-Editor-in-Chief

PLOS Neglected Tropical Diseases
